# The first study on the usefulness of recombinant tetravalent chimeric proteins containing fragments of SAG2, GRA1, ROP1 and AMA1 antigens in the detection of specific anti-*Toxoplasma gondii* antibodies in mouse and human sera

**DOI:** 10.1371/journal.pone.0217866

**Published:** 2019-06-06

**Authors:** Bartłomiej Tomasz Ferra, Lucyna Holec-Gąsior, Justyna Gatkowska, Bożena Dziadek, Katarzyna Dzitko, Weronika Grąźlewska, Dariusz Lautenbach

**Affiliations:** 1 Department of Molecular Biotechnology and Microbiology, Faculty of Chemistry, Gdańsk University of Technology, Gdańsk, Poland; 2 Department of Immunoparasitology, Faculty of Biology and Environmental Protection, University of Lodz, Łódź, Poland; 3 Department of Obstetrics, Medical University of Gdańsk, Gdańsk, Poland; Instituto Butantan, BRAZIL

## Abstract

This study presents an evaluation of four tetravalent recombinant chimeric proteins containing fragments of the *Toxoplasma gondii* antigens, SAG2, GRA1, ROP1 and AMA1, as potential replacements of a the soluble, whole-cell tachyzoite lysate (TLA) used in serological assays. Recombinant chimeric proteins (SAG2-GRA1-ROP1-AMA1N, AMA1N-SAG2-GRA1-ROP1, AMA1C-SAG2-GRA1-ROP1, and AMA1-SAG2-GRA1-ROP1) obtained by genetic engineering were tested for their reactivity with specific IgM and IgG antibodies from sera of experimentally infected mice and humans with *T*. *gondii* infection using an enzyme-linked immunosorbent assay (ELISA). In total 192 serum samples from patients with acquired *T*. *gondii* infection and 137 sera from seronegative individuals were examined. The reactivity of chimeric antigens with antibodies generated during *T*. *gondii* invasion was measured and compared to the results obtained in assays based on whole-cell *Toxoplasma* antigen. Chimeric proteins proved effective in differentiation between *T*. *gondii*-infected and uninfected individuals (100% sensitivity and specificity in the IgG ELISAs) which shows their potential usefulness as a replacements for TLA in standardized commercial tests for the serodiagnosis of toxoplasmosis. In addition, the chimeric proteins were tested for use in avidity determination. Obtained results were comparable to those of the corresponding commercial assays, suggesting the utility of these proteins for avidity assessment. Furthermore, this study demonstrated that the AMA1-SAG2-GRA1-ROP1 chimeric protein has the potential to distinguish specific antibodies from serum samples of individuals with the early and chronic phase of *T*. *gondii* infection.

## Introduction

Toxoplasmosis is a widespread disease caused by the ubiquitous obligate intracellular parasite *Toxoplasma gondii*, which infects a wide range of hosts, including humans [1]. The World Health Organization (WHO) estimates that one-third of the human population is infected with *T*. *gondii*. In healthy individuals, the primary infection is generally asymptomatic or causes relatively mild flu-like symptoms. From the medical point of view, the reliable recognition of *T*. *gondii* invasion is very important in pregnant women, where the significant risk of tachyzoite transmission *via* the placenta to the fetus, can lead to miscarriages or cause neonatal malformations, neurological damage, and blindness in newborns [2,3]. Detection of the parasite invasion is also significant for patients with immunodeficiency, for whom even the chronic phase of toxoplasmosis can be a serious threat. For immunocompromised patients, infection with *T*. *gondii* may cause serious clinical complication, such as fever, headache, encephalitis, pneumonia, myocarditis, conjunctivitis, and nervous system damage [4]. In light of many recent studies, it should be emphasized that the diagnosis of long-acquired *T*. *gondii* infection is also important and may explain the biological basis of some behavioral disorders and neurological diseases. It is assumed that the chronic parasite infection can be associated *inter alia* with the occurrence of seizures, slow response time, memory loss, increased risk of bipolar disorder, schizophrenia and depression, and even suicide rates [5–11].

In routine laboratory diagnostics *T*. *gondii* infection is detected using standard serological assay. Among available serological tests, the enzyme-linked immunosorbent assay (ELISA) has been adapted for the detection of the parasite antigen-specific serum IgG, IgM, and IgA antibodies, and it is widely used. Most commercially available tests employ crude *Toxoplasma* lysate antigens (TLA) which are prepared from *T*. *gondii* tachyzoites propagated in mice or *in vitro* tissue cultures. Despite the high sensitivity and specificity of TLA in immunoassays, the production of these native parasitic antigens is troublesome due to high costs and laborious procedures; moreover, quantification of the antigen mixture, which is a direct result of crude TLA quality, is difficult to standardize. This variation in quality is influenced by diverse culture procedures and methods of lysate preparation between individual laboratories. In addition, ambiguous results obtained from commercial tests can, in some cases, prevent accurate diagnosis. Unfortunately, the appropriate diagnosis can be crucial, especially for pregnant women or immunocompromised people.

Due to difficulties associated with use of crude antigens, new diagnostic tools that can replace TLA in the diagnosis of toxoplasmosis have been sought for many years. The first studies on the possibility of replacing native antigens were proposed in 1991 by Tenter and Johnson, who used two recombinant parasite antigens H4 and H11 [12]. In the following years, many papers describing the production of recombinant *T*. *gondii* antigens in well-characterized expression systems were published [13,14]. As was shown the unquestionable advantage of this approach in antigen production is the ability to easily optimize expression in order to achieve high efficiency. Moreover, this method allows for the use of single proteins or their mixtures which can be used, as selective markers for particular phases of the disease, which is particularly important from a clinical point of view. The use of recombinant antigens improves reproducibility of laboratory results through easy standardization of quantity and composition of the protein preparation.

In recent years, the utility of a new type of diagnostic tool has been demonstrated. Namely, several research teams have obtained recombinant chimeric proteins, which were created from the combination of fragments from two or more genes into a so-called fusion gene. It has been demonstrated that chimeric constructs can be used as diagnostic tools to detect IgG and IgM antibodies, as well as to determine the avidity of IgG antibodies [15–22]. The above works have shown that the advantage of using chimeric proteins is the possibility of a simple standardization of the test, as well as minimization of costs associated with the need to produce and purify each protein separately. Moreover, in comparison to mixtures of recombinant antigens immunoassays based on chimeric proteins were characterized by significantly higher sensitivity and specificity.

The results of our previous research on the recombinant chimeric proteins in serodiagnosis of *T*. *gondii* infection in humans and animals, which are very promising, encouraged us to further work on this topic [19–21,23]. The aim of this study was to improve the performance of the IgM, IgG and IgG avidity ELISA using next generation recombinant chimeric proteins and thus, to demonstrate the diagnostic utility of four *T*. *gondii* recombinant tetravalent chimeric proteins (SAG2-GRA1-ROP1-AMA1N, AMA1N-SAG2-GRA1ROP1, AMA1C-SAG2-GRA1-ROP1, and AMA1-SAG2-GRA1-ROP1), composed of combination of four well-characterized antigens, including dense granule antigen (GRA1), rhoptry antigen (ROP1), surface antigen (SAG2), and different regions of apical membrane antigen (AMA1). Selection of these antigens for the construction of chimeric proteins was based on the earlier immunoassay results, which determined the reactivity of these proteins with specific anti-*T*. *gondii* antibodies developed during the response to naturally-borne invasion. The SAG2 antigen can interact with many cellular surface molecules of infected host [24–27]. The GRA1 antigen plays an important role in the structural modifications of parasitophorous vacuole and is also engaged in strong stimulation of the host immune system [28–33]. The ROP1 protein is an essential player at the early stage of the parasite invasion. This antigen is secreted into the interior of the forming parasitophorous vacuole during parasite entry into host cells, and expression is then inhibited a few hours after invasion [34–36]. AMA1 is a conserved, transmembrane protein secreted from the micronemes of apicomplexan parasites that enable attachment between the parasite surface and the host cell [37–39].

## Materials and methods

### Ethics statement

All experimental procedures were approved and carried out according to guidelines provided by the Polish Local Ethics Commission for Experiments on Animals No. 9 in Lodz (Agreement 75/ŁB639/2012) which operates on the basis of the act issued by the Polish Ministry of Science and Higher Education.

All of the serum samples used in this study were received during routine toxoplasmosis screening from the area of Pomeranian Voivodeship (Poland). The sera were collected as part of the project entitled "Toxoplasmosis—facts and myths. Educational initiative raising social awareness about the infection with protozoan *Toxoplasma gondii*", foundation "Our Children", funded from the Civil Initiatives Fund of the Ministry of Labor and Social Policy (FIO 2008, contract No. 813). Sera were collected from women volunteers for whom standard diagnostic tests were performed, such as the determination of IgG, IgM levels and IgG avidity of anti-*Toxoplasma gondii* antibodies. Information on the obtained anonymized serum samples concerned only the date of collection and the results of anti-*Toxoplasma* antibody tests.

### Parasite

The high-virulence RH *T*. *gondii* strain (ATCC 50174) used for native antigen (TLA) preparation and as a source of DNA template for cloning procedures was maintained *in vitro* on Hs27 (ATCC CRL-1634) cell line in the Iscove’s Modified Dulbecco’s Medium (Cytogen GmbH) supplemented with penicillin (100 U/ml, Sigma-Aldrich), streptomycin (100 μg/ml, Sigma-Aldrich) and fetal calf serum (5%, Cytogen GmbH).

The low-virulence, highly cyst-forming DX strain of *T*. *gondii* used for induction of experimental toxoplasmosis in mice was maintained *in vivo* in laboratory BALB/c mice.

### *Toxoplasma gondii* RNA isolation and cDNA synthesis

Total RNA from tachyzoites was extracted using Total RNA Mini Plus (A&A Biotechnology) according to the manufacturer’s instruction. The isolated RNA was concentrated using Clean-Up RNA Concentrator (A&A Biotechnology) and stored at -80°C. Single strand cDNA was synthesized using iScript cDNA Synthesis Kit (Bio-Rad Laboratories, Inc.) and stored at -20°C.

### Construction of the recombinant plasmids

The DNA encoding AMA1 antigen fragments were amplified from the cDNA, using a standard PCR amplification protocol with the Phusion High-Fidelity DNA Polymerase (Thermo Fisher Scientific, Inc.). The DNA fragments of *ama1* (GenBank: XM_002364813.1) gene were obtained by means of PCR using primers as shown in Table 1. The final PCR products were inserted into the previously obtained recombinant plasmid pET30/SAG2-GRA1-ROP1 [19]. In the case of cloning (*ama1N*) directly after the coding sequence of the ROP1 antigen the PCR product was inserted into the *Sac*I and *Eco*RV sites of the pET30/SAG2-GRA1-ROP1. For cloning (*ama1N*, *ama1C*, and *ama1*) in front of the coding sequence for the SAG2 antigen the PCR products were inserted into *Bgl*II sites of the pET30/SAG2-GRA1-ROP1 using In-Fusion HD Cloning Kit (Takara Bio USA, Inc.).

**Table 1:** Oligonucleotide primers used for the amplification of *ama1* gene fragments.

Gene fragment	Primer name	Primer sequence	Corresponding protein residues
***ama1***	AMA1NC-For AMA1NC-Rev AMA1NN-For AMA1NN-Rev AMA1C-For AMA1C-Rev AMA1NN-For AMA1C-Rev	5’-TGGATCGCAAGCGATCACGTCGGGGAATCCCTTTC-3’ 5’-CAAGCTTGTCGACGGTTGGGCATTTACTGATGAACGCATC TGGG-3’ 5’-TGGACAGCCCAGATCCGTCGGGGAATCCCTTTCAG-3’ 5’-CGCTGGCGTCAGATCTGGGCATTTACTGATGAACGCATCT-3’ 5’-TGGACAGCCCAGATCCAAATCAAGCTCTTCGCGGGTACAG-3’ 5’-CGCTGGCGTCAGATCGTAATCCCCCTCGACCATAACATGT-3’ 5’-TGGACAGCCCAGATCCGTCGGGGAATCCCTTTCAG-3’ 5’-CGCTGGCGTCAGATCGTAATCCCCCTCGACCATAACATGT-3’	AMA1-N 67–287 (221 aa) (add after ROP1) AMA1-N 68–287 (220 aa) (add before SAG2) AMA1-C 287–569 (283 aa) (add before SAG2) AMA1 68–569 (502 aa) (add before SAG2)

The resulting recombinant plasmids contained fusion genes encoding amino acid residues of four different *T*. *gondii* antigens (AMA1, GRA1, ROP1, and SAG2), embedded in frame between the His_6_-tag domains for purification of the recombinant proteins by means of metal affinity chromatography. The amino acid composition of recombinant chimeric tetravalent antigens are in Table 2.

**Table 2:** Plasmid size, amino acid composition and characteristics of the recombinant chimeric tetravalent proteins.

Plasmid (size)	Amino acid residues	Protein characteristic
**pET30/SAG2-GRA1-ROP1-AMA1N** **(7890 bp)**	31–170 SAG2 26–190 GRA1 85–396 ROP1 67–287 AMA1N	892 aa Mw 96.96 kDa pI 5.62
**pET30/AMA1N-SAG2-GRA1-ROP1** **(7911 bp)**	68–287 AMA1N 31–170 SAG2 26–190 GRA1 85–396 ROP1	899 aa Mw 97.95 kDa pI 5.54
**pET30/AMA1C-SAG2-GRA1-ROP1** **(8097 bp)**	287–569 AMA1C 31–170 SAG2 26–190 GRA1 85–396 ROP1	961 aa Mw 103.20 kDa pI 5.00
**pET30/AMA1-SAG2-GRA1-ROP1** **(8757 bp)**	68–569 AMA1 31–170 SAG2 26–190 GRA1 85–396 ROP1	1182 aa Mw 128.42 kDa pI 5.26

### Expression and purification of the chimeric antigens and recombinant proteins

*Escherichia coli* strain Rosetta(DE3)pLacI, transformed with recombinant plasmid pET30/SAG2-GRA1-ROP1-AMA1N or pET30/AMA1N-SAG2-GRA1-ROP1, and *E*. *coli* strain Rosetta(DE3)pLysS, transformed with recombinant plasmids pET30/AMA1C-SAG2-GRA1-ROP1 or pET30/AMA1-SAG2-GRA1-ROP1 were grown in TB media supplemented with 20 μg/ml of kanamycin and 34 μg/ml of chloramphenicol overnight at 23°C. Next, 1000 ml of TB medium, supplemented with the same antibiotics, was inoculated with 20 ml of the overnight culture. The cultures were grown with vigorous shaking at 23°C to the optical density 0.4 at λ = 600 nm (OD_600_). Protein production was then induced with isopropyl-β-D-thiogalactopyranoside (IPTG) at a final concentration of 1 mM, and the recombinant bacteria were incubated with vigorous shaking, for 18 h at the same temperature. The bacterial cells were then harvested by centrifugation. The protein extracts, obtained by dissolving the inclusion bodies (using 8 M urea or 6 M guanidine hydrochloride), were then purified by Ni^2+^-iminodiacetic acid-Sepharose column in accordance with the manufacturer's instructions (Novagen).

Recombinant protein SAG2-GRA1-ROP1 was obtained as previously described [19]. The recombinant proteins were analyzed by means of SDS-PAGE with 10% acrylamide gels and stained with Coomassie blue. The concentrations of the purified proteins were determined by the Bradford method using bovine serum albumin as the standard.

### Preparation of *T*. *gondii* native lysate antigen (TLA)

*Toxoplasma* lysate antigen, serving as a source of parasite’s native antigens, was prepared as described previously [40]. Briefly, the tachyzoites of the *T*. *gondii* RH strain were repeatedly frozen and thawed to lyse the parasite cells. After the centrifugation of resulting cell lysates (20 min at 10,000 g and 4°C) the supernatant containing native *T*. *gondii* antigens (TLA) was stored at -80°C and used in ELISA evaluations. The concentration of proteins in the TLA preparation was determined using Bradford reagent (Sigma-Aldrich) according to the manufacturer’s recommendation.

### Mice

Ten-twenty week-old female BALB/c (naturally high resistant to *T*. *gondii* infection) and C57BL/6 (naturally high susceptible to *T*. *gondii* infection) mice were bred under conventional conditions in the animal facility of the Faculty of Biology and Environmental Protection, University of Lodz. All experimental procedures were carried out according to guidelines provided by the Polish Local Ethics Commission for Experiments on Animals No. 9 in Lodz (Agreement 75/ŁB639/2012).

### Mice infection

BALB/c mice were inoculated intraperitoneally with 5 tissue cysts of the low-virulence *T*. *gondii* DX strain. The parasite cysts used for induction of experimental toxoplasmosis were isolated from the brains of chronically infected C57BL/6 mice by mechanical homogenization and gradient separation, as described previously [41]. Mouse sera were obtained from uninfected animals (negative controls) and infected 2 (acute toxoplasmosis), 3 (late acute toxoplasmosis), 6 and 12 (chronic toxoplasmosis) weeks post infection. Each experimental group consisted of 6 mice.

### IgG and IgM ELISA–mouse serum samples

To determine the levels of specific IgM and IgG antibodies in mouse sera recognizing tested recombinant and control native (TLA) antigens, the immunoenzymatic test was used according to the protocol described previously [41], with minor modifications. Briefly, the serological MaxiSorp plates (Nunc) were coated overnight with the recombinant and native antigens at the optimal concentrations selected during preliminary tests (0.25 μg and 1 μg in 100 μl /well, respectively). For blocking fetal calf serum (10%, Cytogen GmbH) in PBS was used. The control and immune mouse sera were tested at the dilution 1:100 and mouse antibodies were detected using secondary goat anti-mouse IgM and IgG immunoglobulins labeled with horseradish peroxidase (HRP) (Jackson ImunoResearch) at a 1:5000 dilution. The serological reaction was developed using 30% H_2_O_2_ (Sigma-Aldrich) as substrate for HRP and ABTS (Sigma-Aldrich) as chromogene, at the concentration of 1 mg/ml. The absorbance values were estimated at λ = 405 nm using a Multiskan EX ELISA reader (Thermo Fisher Scientific, Inc.). Based on the OD values of controls the cut-off values were calculated as the mean absorbance + 2 standard deviations. Immune sera reactivity with the tested antigens was considered positive if the absorbance exceeded the respective cut-off value.

### Human serum samples

All of the serum samples used in this study were received during routine toxoplasmosis screening from the area of Pomeranian Voivodeship (Poland) and classified as positive or negative based on the results of commercial tests, namely VIDAS TOXO IgM, VIDAS TOXO IgG II and VIDAS TOXO IgG AVIDITY (bioMérieux, Marcy l'Etoile, France), measuring levels of specific anti-*T*. *gondii* IgG and IgM antibodies.

In all tests a total of 329 sera were used, consisting of 64 sera collected from patients with suspected acute phase of *T*. *gondii* infection (IgM +; IgG +; low avidity), 128 sera from patients with chronic *T*. *gondii* infection (IgM +/–; IgG +; high avidity), and 137 sera from healthy individuals (IgM–; IgG–). The pool of sera was divided into appropriate groups and subgroups depending on the performed test based on recombinant antigens.

In the case of the IgG ELISA, the entire available collection of serum samples was used. Group I comprised sera of individuals with suspected acute phase of *T*. *gondii* infection and group II included sera from patients with chronic toxoplasmosis. Moreover, the serum samples from the latter group were further divided into three subgroups; IIA consisted of 32 serum samples with high titers of IgG antibodies (>300 IU/ml), IIB included 32 serum samples with a titer of IgG antibodies between 101 to 300 IU/ml, and IIC consisted of 64 serum samples with low titers of IgG antibodies (≤100 IU/ml). The serum samples of 137 healthy persons not infected with the parasite served as negative control–group III.

In the IgM ELISA test a pool of 207 sera was divided into four experimental groups, namely a) group I consisted of 48 sera with suspected acute phase of *T*. *gondii* infection, b) group II comprised 18 samples of the chronic parasitosis sera with IgM antibodies detectable in the commercial test, c) group III included 58 sera from patients with typical chronic toxoplasmosis and d) control group, IV of 83 serum samples from healthy individuals not infected with *T*. *gondii*.

In order to determine the IgG avidity 60 serum samples were used. Group I comprised 29 serum samples of patients with the suspected acute toxoplasmosis (IgG +; IgM +; low avidity), and group II consisted of 31 sera collected from patients with the chronic phase of *T*. *gondii* infection (IgG +; IgM–; high avidity).

### IgG and IgM ELISA–human serum samples

For IgG ELISA tests MaxiSorp multiwell plates (Nunc) were coated with chimeric proteins SAG2-GRA1-ROP1, SAG2-GRA1-ROP1-AMA1N, AMA1N-SAG2-GRA1-ROP1, AMA1C-SAG2-GRA1-ROP1, and AMA1-SAG2-GRA1-ROP1 or with a TLA at final concentrations of 2.5 μg/ml or 1 μg/ml, respectively, in a coating buffer (0.05 M carbonate buffer, pH 9.6). For IgM ELISA assays MaxiSorp multiwell plates were coated with the same chimeric antigens, or with TLA at final concentrations of 10 μg/ml. After overnight incubation at 4°C, the plates were washed three times with PBS-0.1% Triton X-100 and blocked for 1 h at 37°C in blocking solution (1% bovine serum albumin, 0.5% Triton X-100 in PBS). The wells were then washed three times and incubated for 1 h at 37°C with the human sera diluted 1:100 in blocking solution. Next, the plates were washed three times with washing buffer and incubated with anti-human IgG peroxidase-labeled conjugates (Jackson ImmunoResearch) or anti-human IgM peroxidase-labeled conjugates (Calbiochem) diluted 1:32000 in blocking solution for 1 h at 37°C, followed by the development with o-phenylenediamine dihydrochloride chromogenic substrate (Sigma-Aldrich). After 45 min of incubation at 37°C in darkness, the reaction was stopped by the addition of 0.1 M sulfuric acid and the OD_492_ was measured using a microtiter plate reader (Multiskan FC; Thermo Fisher Scientific, Inc.).

Each serum sample was tested twice. For each sample the results were determined by calculating the mean OD reading of duplicate wells. A positive result was defined as any value higher than the mean OD reading plus 2 standard deviations (cut-off) obtained with 45 (IgG ELISA), or 37 (IgM ELISA) serum samples from the control group of seronegative serum samples.

In the IgG ELISA tests the calculated cut-off values were 0.364 for SAG2-GRA1-ROP1, 0.288 for SAG2-GRA1-ROP1-AMA1N, 0.329 for AMA1N-SAG2-GRA1-ROP1, 0.229 for AMA1C-SAG2-GRA1-ROP1, 0.339 for AMA1-SAG2-GRA1-ROP1, and 0.409 for TLA.

In the IgM ELISA tests calculated cut-off values were 0.465 for SAG2-GRA1-ROP1, 0.305 for SAG2-GRA1-ROP1-AMA1N, 0.483 for AMA1N-SAG2-GRA1-ROP1, 0.433 for AMA1C-SAG2-GRA1-ROP1, 0.329 for AMA1-SAG2-GRA1-ROP1, and 0.947 for TLA.

### IgG avidity ELISA–human serum samples

Two parallel IgG ELISA tests with increasing dilutions of sera (1:50–1:800) were then performed for each reactive serum. One of the tests was conducted as described above and the second contained an additional step; after the incubation with sera, plates were washed three times with 6 M urea buffer (PBS, 0.1% Triton X-100 with 6 M urea). The results were expressed as avidity indexes (AI) calculated as the ratio of optical density for the sample washed with 6 M urea to those obtained without the additional washing step. The serum dilution with OD values between 0.5–1.0 in classical ELISA, without using the 6 M urea solution, was used to calculate the avidity index. An AI below of 0.3 was considered a low avidity, 0.3–0.4 a borderline avidity and values above 0.4 high avidity. This classification was established on the basis of the results obtained and applied previously [33,42,43]. In addition, ROC analysis was performed, on the basis of which a low, borderline, and high avidity indexes were determined using the method described previously [44].

### Statistical analysis

Statistical analysis was performed using SigmaPlot 14.0 software (Systat Software). The Kolmogorov-Smirnov test was applied for assessing the normality of the data distribution. Antibody levels among two groups were compared using t-test and Mann-Whitney U test. ROC analysis was performed to obtain area under the curve (AUC), the sensitivity, and the specificity percentages for the different group of the compared sera. Pearson test was used to analyze correlation between results with recombinant proteins and TLA. Differences with a two-tailed value of *p*<0.05 were considered as statistically significant.

## Results

### Plasmids construction, expression and purification of the recombinant tetravalent chimeric antigens

The nucleotide sequences of the resulting recombinant plasmids were confirmed by DNA sequencing (Genomed, Poland). The size of the expected recombinant plasmids is shown in the Table 2. SAG2-GRA1-ROP1-AMA1N, AMA1N-SAG2-GRA1-ROP1, AMA1C-SAG2-GRA1-ROP1, and AMA1-SAG2-GRA1-ROP1 were expressed as insoluble proteins with a calculated molecular mass between 96.96–128.42 kDa (Table 2), and were purified by one-step metal affinity chromatography. This expression system produces about 11–23 mg of purified proteins per liter of induced culture. Purification resulted in an electrophoretically homogeneous preparation with purity above 90% (results not shown).

### Immunoreactivities of mouse anti-*T*. *gondii* IgM and IgG antibodies in ELISA

**Fig 1:**
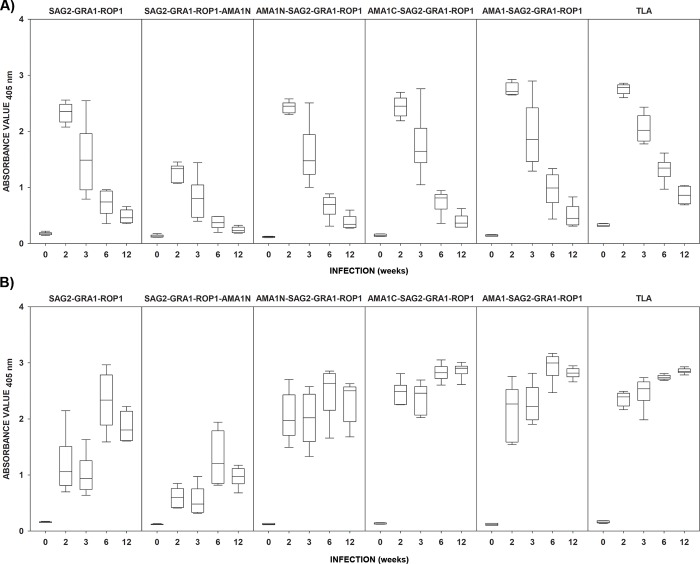
**The antibody response of BALB/c mice infected with *T*. *gondii* DX, tested by IgM ELISA (A) and IgG ELISA (B).** | min-max, ≅ 25%-75%, − median. Uninfected animals were marked as time “0”. The calculated cut-off values were 0.178 (IgG) and 0.227 (IgM) for SAG2-GRA1-ROP1, 0.133 (IgG) and 0.184 (IgM) for SAG2-GRA1-ROP1-AMA1N, 0.150 (IgG) and 0.137 (IgM) for AMA1N-SAG2-GRA1-ROP1, 0.158 (IgG) and 0.187 (IgM) for AMA1C-SAG2-GRA1-ROP1, 0.154 (IgG) and 0.172 (IgM) for AMA1-SAG2-GRA1-ROP1, and 0.202 (IgG) and 0.370 (IgM) for TLA.

As shown in Fig 1, primary invasion of BALB/c mice with DX strain of *T*. *gondii* induced strong humoral IgM and IgG responses to a cocktail of native *T*. *gondii* antigens from TLA preparation. Very similar results were observed for virtually all tested recombinant proteins which were recognized by IgM and IgG immunoglobulins produced in response to experimental *T*. *gondii* invasion. Regardless of the antigen tested the mean reactivity of each immune sera group was higher than that of non-infected controls as shown in S1 Table. During the course of *T*. *gondii* invasion, the reactivity of mouse sera was typically characterized by increasing IgG concentration until 6–12 weeks post-infection, depending on the antigen used, and accompanied by a decrease in IgM immunoglobulins concentration. Although the reactivity of IgM steadily decreased over time, antibodies of this class were still detectable 12 weeks post-infection, using both native and recombinant proteins. All recombinant proteins used displayed 100% IgG reactivity with mouse immune sera regardless of the time post-infection, however the lowest OD values were noted in ELISA using SAG2-GRA1-ROP1-AMA1N as a coating antigen. Of note, use of the AMA1N-SAG2-GRA1-ROP1 fusion protein in the immunoassay resulted in visibly higher reactivity with specific anti-*T*. *gondii* antibodies of both IgM and IgG classes compared to SAG2-GRA1-ROP1-AMA1N. This observation underlines the importance of the position of each individual antigen in the composition of the chimeric construct and its impact on fusion protein reactivity with antibodies generated in response to native parasite antigens during experimental toxoplasmosis.

### IgG ELISA–human serum samples

**Fig 2:**
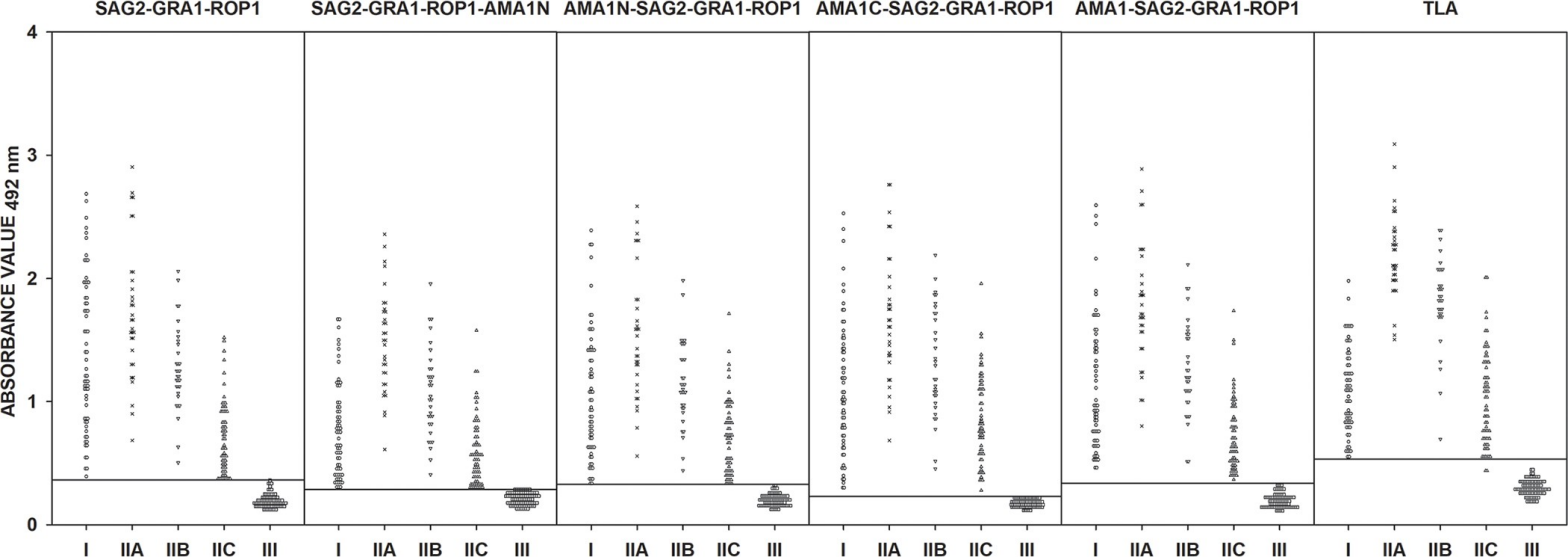
Comparison of immunoreactivity, in IgG ELISA using SAG2-GRA1-ROP1, SAG2-GRA1-ROP1-AMA1N, AMA1N-SAG2-GRA1-ROP1, AMA1C-SAG2-GRA1-ROP1, and AMA1-SAG2-GRA1-ROP1 chimeric proteins, as well as TLA, with sera from patients with suspected acute (I) and chronic *T*. *gondii* infection (IIA–IgG titer >300 IU/ml, IIB–IgG titer between 101–300 IU/ml, and IIC IgG titer ≤100 IU/ml), and from seronegative individuals (III). The horizontal lines represent the cut-off values.

Human serum samples from group I (sera from patients with suspected acute phase of *T*. *gondii* infection), group II (sera from patients in the chronic phase of toxoplasmosis), and group III (sera from seronegative individuals) were examined by IgG ELISA conducted with the SAG2-GRA1-ROP1, SAG2-GRA1-ROP1-AMA1N, AMA1N-SAG2-GRA1-ROP1, AMA1C-SAG2-GRA1-ROP1, and AMA1-SAG2-GRA1-ROP1 recombinant chimeric proteins, and TLA. The 92 sera from group III were used to determine the specificity of the IgG ELISAs. None of these sera were reactive based on the IgG ELISA cut-off value, thereby demonstrating specificity of 100% (Fig 2). The high specificity of our IgG ELISA was confirmed by ROC analysis (Table 3). The calculated cut-off values are very similar to those obtained using the ROC analysis. A decrease in specificity to 97.8% was observed only when chimeric protein SAG2-GRA1-ROP1 was used as a coating antigen. Moreover, we identified a strong positive Pearson correlation between either of the two recombinant chimeric tetravalent proteins, AMA1N-SAG2-GRA1-ROP1 or AMA1-SAG2-GRA1-ROP1, and TLA, 0.637 and 0.578 (*p*<0.001), respectively (Table 4).

**Table 3:** IgG ELISA of recombinant proteins and TLA can discriminate among samples from suspected acute (I) and chronic (II) phase of *T*. *gondii* infection patient groups *vs*. control (III) group.

ANTIGEN	calculated cut-off	ROC cut-off	AUC	Sensitivity, %	Specificity, %
**SAG2-GRA1-ROP1**	0.3641	0.3560	0.9980	100	97.8
**SAG2-GRA1-ROP1-AMA1N**	0.2882	0.2875	1.0000	100	100
**AMA1N-SAG2-GRA1-ROP1**	0.3286	0.3240	1.0000	100	100
**AMA1C-SAG2-GRA1-ROP1**	0.2294	0.2508	1.0000	100	100
**AMA1-SAG2-GRA1-ROP1**	0.3389	0.3478	1.0000	100	100
**TLA**	0.5344	0.4523	0.9999	99.5	100

Serum groups: I–suspected acute *T*. *gondii* infection (IgM +; IgG +; low avidity), *n* = 64. II–chronic *T*. *gondii* infection (IgM–; IgG +; high avidity), *n* = 128. III–control group (IgM–; IgG–), *n* = 92. Sensitivity and specificity were determined using the cut-off value obtained by ROC analysis for the best discrimination.

**Table 4:** Pearson product-moment correlation coefficient (*r*) between results obtained for recombinant proteins *vs*. TLA results for different patient groups.

ELISA test	GROUP	SAG2-GRA1-ROP1		SAG2-GRA1-ROP1-AMA1N		AMA1N-SAG2-GRA1-ROP1		AMA1C-SAG2-GRA1-ROP1		AMA1-SAG2-GRA1-ROP1	
		*r*	*p* value	*r*	*p* value	*r*	*p* value	*r*	*p* value	*r*	*p* value
**IgM**	**I**	0.532	<0.001	0.654	<0.001	0.686	<0.001	0.624	<0.001	0.615	<0.001
	**II**	0.668	0.002	0.900	<0.001	0.614	0.007	0.689	0.002	0.668	0.002
	**III**	0.394	0.002	0.422	<0.001	0.515	<0.001	0.461	<0.001	0.329	0.012
	**IV**	0.714	<0.001	0.686	<0.001	0.551	<0.001	0.617	<0.001	0.753	<0.001
	***All***	0.774	<0.001	0.774	<0.001	0.827	<0.001	0.790	<0.001	0.764	<0.001
**IgG**	**I**	0.879	<0.001	0.796	<0.001	0.823	<0.001	0.915	<0.001	0.816	<0.001
	**IIA**	0.594	<0.001	0.795	<0.001	0.627	<0.001	0.624	<0.001	0.731	<0.001
	**IIB**	0.746	<0.001	0.830	<0.001	0.749	<0.001	0.757	<0.001	0.828	<0.001
	**IIC**	0.722	<0.001	0.762	<0.001	0.608	<0.001	0.582	<0.001	0.784	<0.001
	**IIA-C**	0.854	<0.001	0.901	<0.001	0.813	<0.001	0.793	<0.001	0.895	<0.001
	**III**	0.396	<0.001	0.467	<0.001	0.637	<0.001	0.443	<0.001	0.578	<0.001
	***All***	0.838	<0.001	0.921	<0.001	0.863	<0.001	0.887	<0.001	0.902	<0.001

Serum groups used in IgM ELISA test: I–suspected acute phase of *T*. *gondii* infection (IgM +; IgG +; low avidity), *n* = 48. II–chronic *T*. *gondii* infection with presence of IgM antibodies (IgM +; IgG +; high avidity), *n* = 18. III–chronic *T*. *gondii* infection with absence of IgM antibodies (IgM–; IgG+; high avidity), *n* = 58. IV–control group (IgM–; IgG–), *n* = 83. Serum groups used in IgG ELISA test: I–suspected acute phase of *T*. *gondii* infection (IgM +; IgG +; low avidity), *n* = 64. IIA–chronic *T*. *gondii* infection (IgM–; IgG >300 IU/ml; high avidity), *n* = 32. IIB–chronic *T*. *gondii* infection (IgM–; IgG 101–300 IU/ml; high avidity), *n* = 32. IIC–chronic *T*. *gondii* infection (IgM–; IgG ≤100 IU/ml; high avidity), *n* = 64. IIA-C–chronic *T*. *gondii* infection (IgM–; IgG +; high avidity), *n* = 128. III–control group (IgM–; IgG–), *n* = 137

The sensitivities of the IgG ELISAs based on the SAG2-GRA1-ROP1, SAG2-GRA1-ROP1-AMA1N, AMA1N-SAG2-GRA1-ROP1, AMA1C-SAG2-GRA1-ROP1, and AMA1-SAG2-GRA1-ROP1 recombinant chimeric proteins were calculated as 100% for all of the positive serum samples (Fig 2, Table 3). Using the ROC analysis a lower sensitivity of 99.5% was noted for the IgG ELISA with TLA, as one serum sample from the IIC group (chronic phase of toxoplasmosis, IgG = 14 IU/ml), was not recognized correctly. Analyzing the results obtained for each individual protein preparations, statistically significant differences (*p*<0.001) were found between results from sera of patients with suspected acute and chronic *T*. *gondii* infections compared to the control group (S2 Table). While looking at the results obtained from group I (suspected acute *T*. *gondii* infection), we noted the highest median (1.238) value was obtained from IgG ELISA based on trivalent recombinant chimeric protein SAG2-GRA1-ROP1, which showed a very strong correlation with TLA (*r* = 0.879, *p*<0.001). Although results for all recombinant tetravalent chimeric proteins correlated strongly with TLA (Table 4), the median for individual antigens was lower than for TLA (S2 Table). In group II (chronic phase of *T*. *gondii* infection), the median for the individual subgroups IIA-IIC was highest with TLA, regardless of the IgG antibody titer (S2 Table). However, detailed analysis of the results indicated that addition of AMA1 antigen domain I to the fusion protein did not significantly improve reactivity of the recombinant chimeric protein; in fact, it caused a decrease in the average reads for individual serum samples in the case of high antibody titers. This does not hold true when the second and third domain of AMA1 or the full-length protein (without a signal peptide) were added to the trivalent SAG2-GRA1-ROP1 chimeric protein. Taking into account all statistical analyses (median, ROC analysis, Pearson correlation), the best antigens for detecting IgG antibodies by ELISA are AMA1C-SAG2-GRA1-ROP1 (AUC = 1.000), and AMA1-SAG2-GRA1-ROP1 (AUC = 1.000); (Fig 2; Tables 3 and 4; S2 Table).

### IgM ELISA–human serum samples

**Fig 3:**
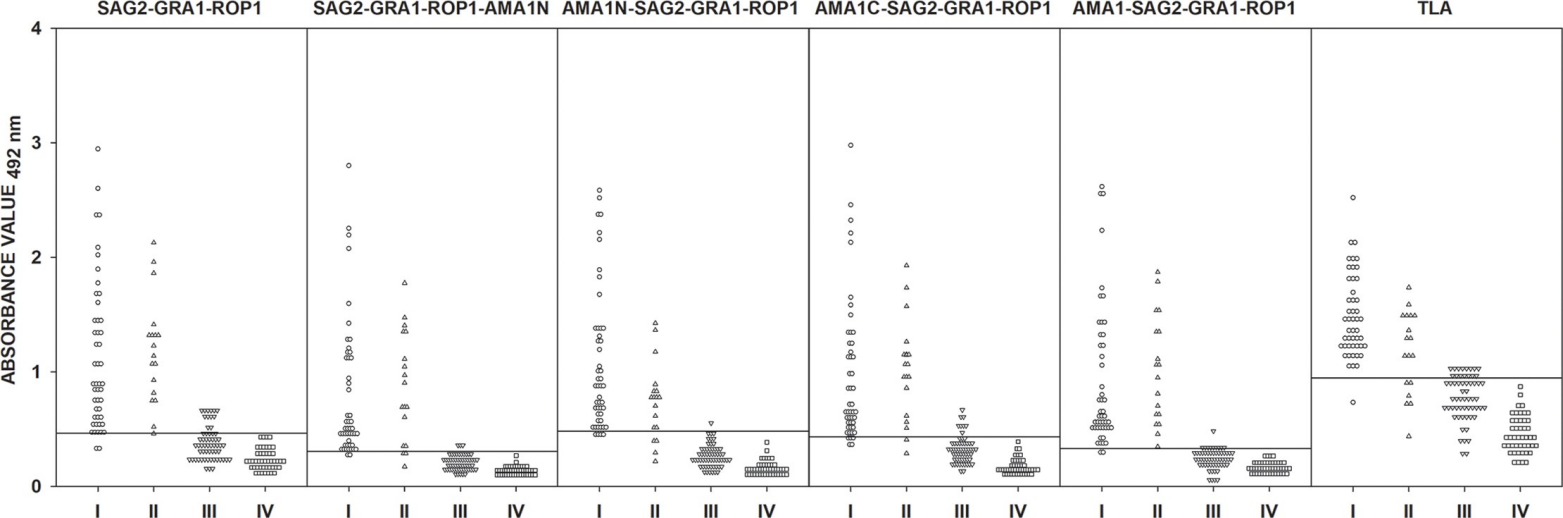
Comparison of immunoreactivity, in IgM ELISA using SAG2-GRA1-ROP1, SAG2-GRA1-ROP1-AMA1N, AMA1N-SAG2-GRA1-ROP1, AMA1C-SAG2-GRA1-ROP1, andAMA1-SAG2-GRA1-ROP1 chimeric proteins, as well as TLA, with sera from patients with suspected acute (I–IgM +, IgG +, low avidity) and chronic *T*. *gondii* infection (II–IgM +, IgG +, high avidity; III–IgM–, IgG +, high avidity), and from seronegative individuals (IV). The horizontal lines represent the cut-off values.

A total of 207 sera from group I, II, III, and IV were examined. The IgM ELISA was conducted using chimeric proteins SAG2-GRA1-ROP1, SAG2-GRA1-ROP1-AMA1N, AMA1N-SAG2-GRA1-ROP1, AMA1C-SAG2-GRA1-ROP1, and AMA1-SAG2-GRA1-ROP1 or TLA. Thirty seven sera from group IV were tested with IgM ELISA in order to calculate the cut-off values. The remaining 46 serum samples, none of which reacted above the cut-off values, were used to determinate the specificity of the assays. In determining assay specificity, results obtained from group III, which included sera taken from patients with chronic *T*. *gondii* infection (IgM–; IgG +; high avidity), were also taken into account. Unfortunately, some of the group III sera reacted above the calculated cut-off values, which resulted in decreased specificity of the IgM ELISA (Fig 3). However, ROC analysis showed that the determined cut-off values were similar for chimeric proteins, and a definite difference was only noted for the TLA preparation (0.947 *vs*. 1.042). Further analyzes involved cut-off values obtained using ROC analysis (Table 5). Taking into account all the results obtained, the highest specificity comparable to TLA (100%) was noted for AMA1N-SAG2-GRA1-ROP1 and AMA1-SAG2-GRA1-ROP1 antigens and it reached 99%. Analyzing the data for individual protein preparations, statistically significant differences (*p*<0.001) were found between the results obtained for serum samples with presence of IgM antibodies (both for suspected acute and chronic phases of *T*. *gondii* infection–group I and II), but also for group III (lack of IgM antibodies) compared to the control group (S2 Table). The sample distribution analysis for AMA1N-SAG2-GRA1-ROP1 and AMA1-SAG2-GRA1-ROP1 chimeric proteins indicates AUC 0.9856 and 0.9942, respectively. Moreover, the results for these antigens showed a strong positive Pearson correlation with TLA, 0.827 and 0.764 (p<0.001), respectively (Table 4). However, only the IgM ELISA using AMA1-SAG2-GRA1-ROP1 displayed increased sensitivity, reaching 95.5% as compared to 87.9% for TLA.

**Table 5:** IgM ELISA using recombinant proteins and TLA can discriminate among samples from different patient groups.

ANTIGEN	ROC analysis	ROC cut-off	AUC	Sensitivity, %	Specificity, %
**SAG2-GRA1-ROP1**	I *vs*. IV I + II *vs*. IV I + II *vs*. III + IV	0.4660	0.9918 0.9941 0.9637	91.7 92.4 92.4	100 100 86.5
**SAG2-GRA1-ROP1-AMA1N**	I *vs*. IV I + II *vs*. IV I + II *vs*. III + IV	0.3060	0.9995 0.9970 0.9842	93.7 90.9 90.9	100 100 97.1
**AMA1N-SAG2-GRA1-ROP1**	I *vs*. IV I + II *vs*. IV I + II *vs*. III + IV	0.4980	1.0000 0.9970 0.9856	89.6 84.9 84.9	100 100 99.0
**AMA1C-SAG2-GRA1-ROP1**	I *vs*. IV I + II *vs*. IV I + II *vs*. III + IV	0.4145	0.9991 0.9980 0.9718	93.8 92.4 92.4	100 100 91.4
**AMA1-SAG2-GRA1-ROP1**	I *vs*. IV I + II *vs*. IV I + II *vs*. III + IV	0.3560	1.0000 1.0000 0.9942	95.8 95.5 95.5	100 100 99.0
**TLA**	I *vs*. IV I + II *vs*. IV I + II *vs*. III + IV	1.0420	0.9991 0.9895 0.9626	95.8 87.9 87.9	100 100 100

Serum groups: I–suspected acute phase of *T*. *gondii* infection (IgM +; IgG +; low avidity), *n* = 48. II–chronic *T*. *gondii* infection with presence of IgM antibodies (IgM +; IgG +; high avidity), *n* = 18. III–chronic *T*. *gondii* infection with absence of IgM antibodies (IgM–; IgG +; high avidity), *n* = 58. IV–control group (IgM–; IgG–), *n* = 46. AUC was determined for the group or groups clusters indicated in the second column. Sensitivity and specificity were determined using the cut-off value obtained by ROC analysis (for each antigen preparation the same cut-off value was adopted for all compared groups–as a result, a lower sensitivity was obtained for some compared groups), for the best discrimination among the groups indicated in each row.

### IgG avidity–human serum samples

**Fig 4:**
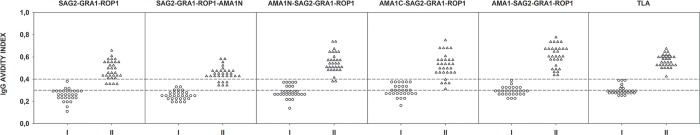
Avidity indexes obtained for sera from patients with suspected acute (I–IgM +, IgG +, low avidity) and chronic phase of *T*. *gondii* infection (II–IgM–, IgG +, high avidity) with SAG2-GRA1-ROP1, SAG2-GRA1-ROP1-AMA1N, AMA1N-SAG2-GRA1-ROP1, AMA1C-SAG2-GRA1-ROP1, AMA1-SAG2-GRA1-ROP1 chimeric proteins and TLA. The horizontal lines represent the borderline values of avidity indexes (0.3 and 0.4).

The ability of recombinant chimeric proteins to differentiate between serum samples with low and high avidity index was assessed in two ways. The first method was grounded on the assumption that borderline avidity was in the range between 0.3–0.4, while the second was based on ROC analysis. In the first case, results showed large variation in avidity indexes obtained for individual chimeric proteins (Fig 4, S3 Table, Table 5). From chronic phase *T*. *gondii* infected serum samples, only one protein, AMA1-SAG2-GRA1-ROP1, allowed for the correct determination of the avidity index as high. The borderline index of avidity was determined for other antigens for some sera (6.5–19.4%) (S3 Table). This sharply contrast results from suspected acute phase *T*. *gondii* infected sera, in which low avidity was determined correctly for 89.7% when SAG2-GRA1-ROP1 and SAG2-GRA1-ROP1-AMA1N proteins were used. However, results obtained for TLA (69%) were comparable to those obtained for recombinant protein AMA1-SAG2-GRA1-ROP1 (65.5%).

Using the second method, a ROC analysis was performed; after calculating the cut-off value, it was assumed that borderline avidity falls between cut-off values of ±10%. As shown in Table 6, the range of values for which sera can be classified as having borderline avidity varies depending on the antigen used. In addition, it is easier to qualify sera with both low and high avidity. The sample distribution analysis indicates AUC values of 1.000, 0.9989, 0.9978, 0.9956 and 0.9700 for AMA1-SAG2-GRA1-ROP1 or TLA, SAG2-GRA1-ROP1-AMA1N, AMA1N-SAG2-GRA1-ROP1, SAG2-GRA1-ROP1, and AMA1C-SAG2-GRA1-ROP1, respectively. As was also the case with our first method, the chimeric protein, AMA1-SAG2-GRA1-ROP1, showed the greatest potential for determining avidity of IgG antibodies, and was characterized by a 100% sensitivity and specificity compared to the TLA.

**Table 6:** IgG avidity ELISA assays using recombinant proteins and TLA to discriminate among samples from suspected acute (I) *vs*. chronic (II) phase of *T*. *gondii* infection patient groups.

ANTIGEN	ROC cut-off	Undetermined zone “borderline AI”	Avidity index (AI)		AUC	Sensitivity, %	Specificity, %
			GROUP I (n = 29)	GROUP II (n = 31)			
**SAG2-GRA1-ROP1**	0.3325	0.2993–0.3656	L: 26 **B: 2** **H: 1**	L: 0 **B: 3** H: 28	0.9956	100	96.6
**SAG2-GRA1-ROP1-AMA1N**	0.3433	0.3090–0.3776	L: 27 **B: 2** H: 0	L: 0 **B: 6** H: 25	0.9989	96.8	100
**AMA1N-SAG2-GRA1-ROP1**	0.3882	0.3494–0.4270	L: 24 **B: 5** H: 0	L: 0 **B: 3** H: 28	0.9978	96.8	100
**AMA1C-SAG2-GRA1-ROP1**	0.3862	0.3476–0.4248	L: 22 **B: 7** H: 0	L: 0 **B: 5** H: 26	0.9700	87.1	100
**AMA1-SAG2-GRA1-ROP1**	0.4068	0.3661–0.4475	L: 28 **B: 1** H: 0	L: 0 **B: 2** H: 29	1.0000	100	100
**TLA**	0.4102	0.3692–0.4512	L: 26 **B: 3** H: 0	L: 0 **B: 1** H: 30	1.0000	100	100

Serum groups: I–suspected acute phase of *T*. *gondii* infection (IgM +; IgG +; low avidity), *n* = 29. II–chronic *T*. *gondii* infection (IgM–; IgG +; high avidity), *n* = 31. AI–avidity index; L–low avidity index; B–borderline avidity index; H–high avidity index. Sensitivity and specificity were:—determined using the cut-off value obtained by ROC analysis for the best discrimination.—calculated taking into account samples displaying AI values out of the undetermined zone

## Discussion

Currently serological tools such as ELISA and immunoblots are commonly used in diagnosing *T*. *gondii* infection. Progress in research methodology has allowed well-purified recombinant proteins to be obtained *via* molecular biology that are attractive alternatives for the detection of serum antibodies compared to commercial tests based mainly on TLA lysate. In our study, for the first time, four new tetravalent recombinant chimeric antigens of *T*. *gondii* (SAG2-GRA1-ROP1-AMA1N, AMA1N-SAG2-GRA1ROP1, AMA1C-SAG2-GRA1-ROP1, and AMA1-SAG2-GRA1-ROP1), were expressed and purified, and their performance was evaluated in the detection of specific IgG and IgM antibodies in human and mouse sera.

Until now, there were only a few studies demonstrating the usefulness of the divalent and trivalent recombinant chimeric proteins in the detection of specific anti-*T*. *gondii* antibodies. The first study, involved two antigens composed of the most immunoreactive regions of the antigens MIC2_157-235_, MIC3_234-307_, SAG1_182-312_ (GST-EC2) and GRA3_36-134_, GRA7_24-102_, M2AP_37-263_ (GST-EC3) [15]. The sensitivity of IgG ELISA for both EC2 and EC3 proteinss was estimated at 100%. It has been demonstrated that these chimeric proteins can also be used successfully for the detection of IgM antibodies in the sera of patients with the early phase of *T*. *gondii* infection; EC2 and EC3 antigens were characterized by 98%, and 84% reactivity, respectively. Another antigen used in the detection of IgG and IgM immunoglobulins in human sera was the SAG1/2 chimeric protein [22]. Western blot assays revealed the utility of this antigen to detect infection in patients with early acute, acute and chronic toxoplasmosis. Dai et al. [16,17] developed a recombinant multiepitope fusion protein (rMEP) composed of three antigenic determinants: SAG1_309-318_, SAG2_109-118_, and SAG3_347-356_ recognized by B-lymphocytes. The rMEP protein was found to be highly reactive in IgG ELISA tests of serum samples from both early (87.5%) and chronic (97.4%) *T*. *gondii* infection. In addition, the sensitivity of the IgM ELISA based on the rMEP antigen reached 96.9%, which suggested that this antigen may be important in differentiating between the early and chronic phase of the disease. This capacity to distinguish early from chronic *T*. *gondii* infection was confirmed in the next paper, which showed that the ability to distinguish infection phase was dependent on choosing the optimal concentration of rMEP antigen for the IgG and IgM ELISA assays. The sensitivity of the IgG ELISA was estimated at 25.9% for sera from the early phase of *T*. *gondii* infection and 97.1% for sera representing chronic phase of the disease, whereas the sensitivity of the IgM ELISA test was 96.6%. Nevertheless, the authors did not report the number of sera used to determine the cut-off value, and a relatively small pool of serum samples were used to determine the specificity of the test. In our previous studies, we also showed the usefulness of divalent and trivalent recombinant chimeric proteins for diagnosis of toxoplasmosis [18–21,23]. We found that the chimeric proteins are characterized by high reactivity similar to native polyvalent TLA antigen, and markedly higher than individual proteins or mixtures of recombinant proteins [19–21]. Our previous findings have shown that during the construction of chimeric proteins, particular attention should be paid not only to the number and size of immunodominant fragments of individual antigens, but also to their order in the final construct. The results of all previously described works show that recombinant chimeric proteins may be an attractive alternative to TLA used in commercial serological assays to detect anti-*T*. *gondii* IgG and IgM antibodies. The use of recombinant chimeric proteins in such assays yields improved reproducibility, easier standardization, and the ability to consistently choose the same composition of the protein preparation.

The aim of this study was to improve the performance of the IgM, IgG and IgG avidity ELISA using next generation recombinant chimeric proteins and thus, to demonstrate the diagnostic utility of four *T*. *gondii* recombinant tetravalent chimeric proteins, composed of combination of four well-characterized antigens: GRA1, ROP1, SAG2, and different regions of AMA1. In addition their diagnostic potential as compared to the previously tested SAG2-GRA1-ROP1 protein [19], and commercially available tools for determination of *T*. *gondii* infection.

The present study documents, for the first time, assessment of humoral immune response dynamics during murine experimental toxoplasmosis using tetravalent recombinant chimeric proteins. Our results indicate that all recombinant chimeric proteins enable determination of a strong dynamic of induced immune response, involving the synthesis of antigen-specific IgM antibodies, which appear and achieve maximal levels in the acute stage of *T*. *gondii* invasion. A substantial decrease in IgM antibodies can be observed in third week after infection, when acute infection transitions to the chronic phase. At this point in the parasite infection, production of IgM and IgG class antibodies are switched, with IgG antibodies becoming the predominantly synthesized. This has been observed in particular for the recombinant tetravalent chimeric proteins AMA1N-SAG2-GRA1-ROP1, AMA1C-SAG2-GRA1-ROP1, and AMA1-SAG2-GRA1-ROP1. The synthesis of IgM and IgG immunoglobulins, which recognize these antigens, is most similar to that noted with TLA, used for comparative purposes. In contrast SAG2-GRA1-ROP1 and SAG2-GRA1-ROP1-AMA1 chimeric proteins exhibited the strongest reactivity with IgG antibodies 6 weeks post-infection. Moreover, the addition of an AMA1N antigen fragment at the C-terminus of the SAG2-GRA1-ROP1 protein resulted in significantly decreased reactivity with both IgM and IgG antibodies. Analyzing the results for recombinant tetravalent chimeric proteins containing an AMA1 fragment on the N-terminus of the SAG2-GRA1-ROP1, we observed a significant increase in detection of IgG antibodies. This may be due to the fact that the AMA1 antigen is a marker characteristic of the early phase of the toxoplasmosis, and, together with the RONs antigens, plays a pivotal role in the initial stages of invasion into the host cell. On the other hand, scrutinizing the results from SAG2-GRA1-ROP1-AMA1N in comparison to AMA1N-SAG2-GRA1-ROP1, AMA1C-SAG2-GRA1-ROP1, and AMA1-SAG2-GRA1-ROP1 proteins, implies that fragment position may decrease epitope exposure. These results confirm our earlier observations, related to the construction of recombinant chimeric proteins, which showed that not only the size of immunodominant fragments [19], but also their order in the final construct is important. It is commonly known that recombinant proteins produced in a bacterial expression system, such as *E*. *coli*, often lose their antigenic properties due to incorrect folding. Furthermore, recombinant chimeric proteins are not naturally occurring components; it is possible that some fragments of immunodominant regions interact with each other and/or the amino acid sequence of linked antigens does not allow for creation of all functional epitopes. For this reason, some characteristic epitopes of native antigens are not present in the recombinant protein and therefore cannot be recognized by specific antibodies. On the other hand, during the construction of chimeric proteins, we must not create additional hydrophobic domains which can be recognized by specific antibodies. We excluded this possibility for presented chimeric constructs through analysis using bioinformatics software. Summarizing our results from the murine toxoplasmosis model, we can conclude, that recombinant chimeric proteins hold great promise for detecting specific early IgM, a potential serological marker of acute *T*. *gondii* infection, and IgG antibody regardless of the phase of *T*. *gondii* infection.

In case of human serum samples, our findings showed sensitivity and specificity of 100% for all IgG ELISAs with tetravalent chimeric proteins in comparison to result from the previously described trivalent SAG2-GRA1-ROP1 protein [19], and commercially used TLA. Statistical analysis shows that recombinant chimeric proteins are an attractive alternative to TLA for the detection of IgG antibodies, with the use of a completely new pool of sera generating results comparable to those of previous studies. There was only a slight decrease in the specificity of the test for the previously described SAG2-GRA1-ROP1 protein (100% *vs*. 97.8%). However, our overall results confirm the high diagnostic potential of recombinant fusion proteins in IgG ELISAs. Interestingly, variability in IgG ELISAs results from human serum samples, noted for individual chimeric proteins, are definitely less pronounced than those from the murine model of toxoplasmosis. However, consistent findings, such as lower reactivity of the SAG2-GRA1-ROP1-AMA1N chimeric protein containing the AMA1N fragment at the C-terminus, were also observed. Moreover, among fusion proteins containing N-terminal AMA1 antigen fragments–AMA1C-SAG2-GRA1-ROP1, and AMA1-SAG2-GRA1-ROP1, were more reactive than AMA1N-SAG2-GRA1-ROP1. These results confirm the earlier hypothesis that for the SAG2-GRA1-ROP1-AMA1N chimeric construct antigenic determinants are likely less prominent.

The chimeric proteins were constructed based on the assumption that they could be used to detect antigen-specific IgG antibodies in a precise phase of *T*. *gondii* infection. Unfortunately, achieving this goal is very arduous, which can be explained by the example of recombinant proteins ROP1 and MAG1. Both of these antigens are potential selection markers for distinguishing acute and chronic *T*. *gondii* infection. If we use the ROP1 and MAG1 antigens in the IgG ELISA, it allows for the detection of IgG antibodies in sera from the acute phase of *T*. *gondii* infection at 94.6% and 97.3% sensitivity, and at 15.5% and 7.5% in chronic phase sera [36,45]. Our previous research has shown that the combination of these two proteins in the recombinant chimeric MAG1-ROP1 protein resulted in a decrease in the ability to differentiate between the phases of the disease–sensitivity of the IgG ELISA with the use of serum samples from acute and chronic phase of *T*. *gondii* infection was 86.4% and 32.8%, respectively [18]. Therefore, further research is necessary to identify ideal tool for detecting IgG antibodies alone, and exclusively at a particular stage of the disease. However, detection of IgM antibodies and testing maturation of IgG antibodies may allow solving this problem. This is mainly due to the fact that the development of IgM and IgG antibody classes are switched during parasitosis [46–48]. Specifically IgM antibodies disappear relatively quickly after the initial invasion, and they are not associated with immune memory. In addition, in the case of IgG antibodies, we observe a genetically determined phenomenon of the antibody maturation, which is associated with an increase in specific binding to the antigen and may look different between individuals. The findings of our study showed very high sensitivity and specificity of IgM ELISAs with tetravalent chimeric proteins. Of all the chimeric constructs tested, the AMA1-SAG2-GRA1-ROP1 protein is characterized by the highest reactivity (95.5%) in the detection of IgM antibodies. Moreover, the specificity of the IgM ELISA based on this chimeric protein is 99% and is comparable to the TLA test. Lower specificity can be explained by the detection of late IgM, but in our opinion, it results rather from non-specific interaction with IgG antibodies. However, it should be remembered that commercially available tests for detection of IgM antibodies are based on capture “sandwich” ELISA, which is more specific. In the case of the sensitivity of tests for both IgM and IgG ELISA, it should be emphasized that the reactivity of antigens is closely related to the composition of the immunodominant regions in the final constructs. The reactivity of chimeric proteins is also influenced by the size of the immunodominant fragment, as we observe definitively higher reactivity in tetravalent chimeric proteins containing the AMA1C (C-terminal fragment of the AMA1 protein), and AMA1 protein (full-length AMA1 without a signal peptide). We observed a similar situation in the case of the SAG2-GRA1-ROP1 chimeric construct containing a fragment of ROP1 corresponding to amino acid residues 85–396, which contains a part of the alkaline C-terminal region rich in arginine, and is likely to be crucial for antigen recognition by the specific antibodies [19]. The high reactivity of the SAG2-GRA1-ROP1 chimeric protein can be also explained by another hypothesis. The C-terminal region of ROP1 may contain at least one immunodominant B-cell epitope recognized by the majority of individuals, whereas responses to N-terminal region of ROP1 are much more variable [23]. The obtained results are also consistent with our research on the AMA1 protein where we have shown that only the C-terminal fragment of AMA1 and the full-length AMA1 are well recognized by IgM and IgG antibodies (paper under review). As is known AMA1 antigen together with rhoptry neck proteins (RONs) forms a structural complex called the moving junction, which migrates from the anterior to the posterior of the parasite, leading to the internalization of the parasite into a parasitophorous vacuole [49]. In the entire complex AMA1 protein is a bridge between the surface of the parasite cell and RONs proteins that interact with the surface of the host cell. Analyzing the structure of the AMA1 protein, we can conclude that the N-terminal fragment of the AMA1 (domain I) is responsible for the binding of the RON2 antigen, while the C-terminal fragments (domain II and III) remains unbound to other proteins [50,51]. This structure of the AMA1 protein suggests that there is a high probability that the C-terminal fragment of the protein is better recognized by specific antibodies, since it was shown that the fragment of the AMA1 antigen corresponding to the amino acid residues from 263 to 457 includes at least one B-cell epitope [52]. The IgG ELISA assay demonstrated that this antigen is capable of detecting IgG antibodies in serum samples from healthy adults with acquired infection (78%), and in serum samples from children with congenital infection (60%).

Despite many years of research on recombinant antigens, to date, no protein has been found and used in practice, which would be a selective marker allowing for differentiation between the acute and chronic phase of *T*. *gondii* infection. However, many studies have demonstrated the potential diagnostic utility of recombinant proteins to detect anti-*T*. *gondii* IgM or IgG antibodies [13,14]. Taking into account everything above, it seems that since the ideal selection marker of the acute and chronic phase of *T*. *gondii* infection has not been found so far, it is necessary to determine the dynamic of maturation of IgG antibodies. To date, only a few papers can be found in the literature regarding the use of recombinant proteins to determine the avidity of IgG antibodies [33,43,44,53]. We believe this is mainly due to difficult interpretation of results from such experiments, based on a number of assumptions that must be made at the beginning of the assay. In the IgG ELISA avidity test, we need to determine in the ranges for obtained results that indicate low, borderline or high avidity. Although every commercially available test contains a description of how the results should be interpreted, some works presuppose that borderline avidity for recombinant proteins should be in between 0.3–0.4 [33,42,43]. However, in view of the results presented by Costa et al., [44], it seems that determination of borderline avidity is dependent on the specificity of antigen used in the test. In our work, we described both ways of interpreting results from avidity assessment. Regardless of the interpretation method, the results allowed us to draw similar conclusions, though determination of the IgG avidity index based on ROC analysis was more reliable. The model proposed by Costa et al., [44], includes the possibility of a diversified response to specific antigens against which anti-*T*. *gondii* antibodies are directed. Regardless of the way the results are interpreted, the AMA1-SAG2-GRA1-ROP1 protein results in values comparable to the commercial test and TLA used for comparative purposes. Our findings using sera samples taken from the same patients at different intervals showed that regardless of the IgG antibody titer, maturation increases over time (S4 Table). This property of the specific chimeric proteins raises their potential significance for the diagnosis of *T*. *gondii* infection. It is known that low avidity index should be present for the first 4 months after the onset of the infection, but in the case of sera collected from one patient, the low avidity of IgG persisted even after 23 weeks from the first collection (unknown exact moment of infection) [46,54]. It should be remembered that in the case of pregnant women, after the detection of primary infection, pharmacological therapy is included to prevent fetal damage or miscarriage. However, such therapy may result in longer persistence of IgM antibodies and slower maturation of IgG antibodies [55–57]. Unfortunately, it is very difficult to obtain human sera, for which we know the exact moment of infection, and even more so from pregnant women who will not be treated in the event of the primary infection.

To summarize, this report presents results that demonstrate, for the first time, that tetravalent recombinant chimeric proteins can be successfully used in the serodiagnosis of *T*. *gondii* infection. Results obtained in IgM, IgG and IgG avidity ELISAs based on chimeric proteins yield data comparable to TLA, used in commercial assays. Similar to our previous papers, it was shown that during the construction of chimeric proteins particular attention should be paid to the size of antigen fragments, but also to their order in the final fusion construct. For the first time, IgM and IgG antibodies produced during the progress of *T*. *gondii* infection were analyzed using a mouse model of toxoplasmosis. The results of the study with human sera demonstrated, that a newly produced tetravalent chimeric proteins can be a good diagnostic tool for detecting IgG antibodies. Furthermore, one of the produced chimeric proteins, namely AMA1-SAG2-GRA1-ROP1, possesses a high ability to detect IgM antibodies and to determine the avidity index; therefore, it can be used to differentiate between the acute and chronic toxoplasmosis. Nevertheless, before utilizing the AMA1-SAG2-GRA1-ROP1 chimeric protein instead of the TLA in the clinical diagnostic of *T*. *gondii* infection more assays using a larger pool of sera and involving other research laboratories are required. However, it should be highlighted that the present results are very promising.

## Supporting information

S1 Table The analysis of IgM and IgG antibody levels in the progress of *T*. *gondii* infection in BALB/c mice. (DOCX)

S2 Table The analysis of IgM and IgG antibody levels in the different serum groups. Serum groups used in IgM ELISA test:
I–suspected acute phase of *T*. *gondii* infection (IgM +; IgG +; low avidity), *n* = 48
II–chronic *T*. *gondii* infection with presence of IgM antibodies (IgM +; IgG +; high avidity), *n* = 18
III–chronic *T*. *gondii* infection with absence of IgM antibodies (IgM–; IgG +; high avidity), *n* = 58
IV–control group (IgM–; IgG–), *n* = 83
Serum groups used in IgG ELISA test:
I–suspected acute phase of *T*. *gondii* infection (IgM +; IgG +; low avidity), *n* = 64
IIA–chronic *T*. *gondii* infection (IgM–; IgG >300 IU/ml; high avidity), *n* = 32
IIB–chronic *T*. *gondii* infection (IgM–; IgG 101–300 IU/ml; high avidity), *n* = 32
IIC–chronic *T*. *gondii* infection (IgM–; IgG ≤100 IU/ml; high avidity), *n* = 64
IIA-C–chronic *T*. *gondii* infection (IgM–; IgG +, high avidity), *n* = 128
III–control group (IgM–; IgG–), *n* = 137.
(DOCX)

S3 Table Comparison of a commercial avidity test and an IgG avidity ELISA tests with recombinant proteins and TLA based on results for sera from patients in the suspected acute (I) or chronic (II) phase of *T*. *gondii* infection. Serum groups:
I–suspected acute phase of *T*. *gondii* infection (IgM +; IgG +; low avidity), *n* = 29
II–chronic *T*. *gondii* infection (IgM–; IgG +; high avidity), *n* = 31
Explanation:
AI–avidity index
L–low avidity index
B–borderline avidity index
H–high avidity index
For commercial test VIDAS TOXO IgG AVIDITY (bioMérieux, Marcy l'Etoile, France), an AI below 0.2 is low, an AI of 0.2–0.3 is borderline, and an AI over 0.3 is high; for recombinant chimeric proteins and TLA, an AI below 0.3 is low, an AI of 0.3–0.4 is borderline, and an AI over 0.4 is high.
(DOCX)

S4 Table Comparison of IgM, IgG, and IgG avidity maturation of commercial tests results with ELISA’s tests results using recombinant proteins with serum samples obtained from pregnant women suspected of acute phase of *T*. *gondii* infection taken at various time intervals. Explanation:
IgM: (+)–positive result; (–)–negative result (the result below the cut-off value calculated for the specific antigen preparation)IgG avidity maturation: AI–avidity index; L–low avidity index; B–borderline avidity index; H–high avidity index.* the result includes the repetition resulting from the so-called gray zone
IgM, IgG, and IgG avidity maturation on the basis of a ROC analysis.
Results marked in bold–incompatible with the results of a commercial test.
(DOCX)
