# Supplementary material for: The first study on the usefulness of recombinant tetravalent chimeric proteins containing fragments of SAG2, GRA1, ROP1 and AMA1 antigens in the detection of specific anti-Toxoplasma gondii antibodies in mouse and human sera
Source: PLoS One. 2019 Jun 6;14(6):e0217866. doi: 10.1371/journal.pone.0217866 (PMC6553757; doi:10.1371/journal.pone.0217866)
Supplement: S1 Table — (DOCX) [file pone.0217866.s001.docx]

**S1 Table.** **The analysis of IgM and IgG antibody levels in the progress of *T. gondii* infection in BALB/c mice.**

| **ANTIGEN** | **Time after challenge** | **IgM level** | | **IgG level** | |
| --- | --- | --- | --- | --- | --- |
|  |  | **Median** | ***p* value** | **Median** | ***p* value** |
| **SAG2-GRA1-ROP1** | **0**  **2**  **3**  **6**  **12** | 0.173  2.354  1.486  0.740  0.459 | -  <0.001  0.002  <0.001  <0.001 | 0.153  1.059  0.940  2.336  1.0803 | -  0.002  0.002  <0.001  0.002 |
| **SAG2-GRA1-ROP1-AMA1N** | **0**  **2**  **3**  **6**  **12** | 0.134  1.333  0.799  0.370  0.229 | -  0.002  0.002  <0.001  0.002 | 0.117  0.600  0.480  1.204  0.974 | -  <0.001  0.002  0.002  0.002 |
| **AMA1N- SAG2-GRA1-ROP1** | **0**  **2**  **3**  **6**  **12** | 0.117  2.450  1.475  0.694  0.343 | -  0.002  0.002  <0.001  0.002 | 0.121  1.974  2.019  2.633  2.501 | -  0.002  0.002  0.002  0.002 |
| **AMA1C-SAG2-GRA1-ROP1** | **0**  **2**  **3**  **6**  **12** | 0.143  2.452  1.644  0.812  0.365 | -  <0.001  0.002  0.002  <0.001 | 0.131  2.490  2.458  2.823  2.906 | -  0.002  0.002  0.002  0.002 |
| **AMA1- SAG2-GRA1-ROP1** | **0**  **2**  **3**  **6**  **12** | 0.147  2.715  1.856  0.992  0.451 | -  0.002  0.002  0.002  0.002 | 0.115  2.267  2.225  3.000  2.818 | -  0.002  0.002  0.002  <0.001 |
| **TLA** | **0**  **2**  **3**  **6**  **12** | 0.318  2.784  2.018  1.343  0.862 | -  <0.001  0.002  0.002  <0.001 | 0.159  2.392  2.539  2.732  2.846 | -  <0.001  0.002  <0.001  <0.001 |
