# Supplementary material for: The first study on the usefulness of recombinant tetravalent chimeric proteins containing fragments of SAG2, GRA1, ROP1 and AMA1 antigens in the detection of specific anti-Toxoplasma gondii antibodies in mouse and human sera
Source: PLoS One. 2019 Jun 6;14(6):e0217866. doi: 10.1371/journal.pone.0217866 (PMC6553757; doi:10.1371/journal.pone.0217866)
Supplement: S2 Table — Serum groups used in IgM ELISA test: I–suspected acute phase of T. gondii infection (IgM +; IgG +; low avidity), n = 48 II–chronic T. gondii infection with presence of IgM antibodies (IgM +; IgG +; high avidity), n = 18 III–chronic T. gondii infection with absence of IgM antibodies (IgM–; IgG +; high avidity), n = 58 IV–control group (IgM–; IgG–), n = 83 Serum groups used in IgG ELISA test: I–suspected acute phase of T. gondii infection (IgM +; IgG +; low avidity), n = 64 IIA–chronic T. gondii infection (IgM–; IgG >300 IU/ml; high avidity), n = 32 IIB–chronic T. gondii infection (IgM–; IgG 101–300 IU/ml; high avidity), n = 32 IIC–chronic T. gondii infection (IgM–; IgG ≤100 IU/ml; high avidity), n = 64 IIA-C–chronic T. gondii infection (IgM–; IgG +, high avidity), n = 128 III–control group (IgM–; IgG–), n = 137. (DOCX) [file pone.0217866.s002.docx]

**S2 Table. The analysis of IgM and IgG antibody levels in the different serum groups.**

| **ANTIGEN** | **IgM** | | | **IgG** | | |
| --- | --- | --- | --- | --- | --- | --- |
|  | **GROUP** | **Median** | ***p* value** | **GROUP** | **Median** | ***p* value** |
| **SAG2-GRA1-ROP1** | **I**  **II**  **III**  **IV** | 0.887  1.184  0.365  0.220 | <0.001  <0.001  <0.001  - | **I**  **IIA**  **IIB**  **IIC**  **IIA-C**  **III** | 1.238  1.663  1.221  0.663  0.994  0.184 | <0.001  <0.001  <0.001  <0.001  <0.001  - |
| **SAG2-GRA1-ROP1-AMA1N** | **I**  **II**  **III**  **IV** | 0.500  0.809  0.200  0.130 | <0.001  <0.001  <0.001  - | **I**  **IIA**  **IIB**  **IIC**  **IIA-C**  **III** | 0.721  1.497  1.033  0.532  0.865  0.217 | <0.001  <0.001  <0.001  <0.001  <0.001  - |
| **AMA1N-SAG2-GRA1-ROP1** | **I**  **II**  **III**  **IV** | 0.877  0.768  0.245  0.147 | <0.001  <0.001  <0.001  - | **I**  **IIA**  **IIB**  **IIC**  **IIA-C**  **III** | 0.945  1.481  1.115  0.706  0.975  0.195 | <0.001  <0.001  <0.001  <0.001  <0.001  - |
| **AMA1C-SAG2-GRA1-ROP1** | **I**  **II**  **III**  **IV** | 0.685  1.020  0.312  0.154 | <0.001  <0.001  <0.001  - | **I**  **IIA**  **IIB**  **IIC**  **IIA-C**  **III** | 1.019  1.636  1.242  0.800  1.112  0.167 | <0.001  <0.001  <0.001  <0.001  <0.001  - |
| **AMA1-SAG2-GRA1-ROP1** | **I**  **II**  **III**  **IV** | 0.621  0.998  0.243  0.157 | <0.001  <0.001  <0.001  - | **I**  **IIA**  **IIB**  **IIC**  **IIA-C**  **III** | 0.942  1.711  1.201  0.638  1.015  0.187 | <0.001  <0.001  <0.001  <0.001  <0.001  - |
| **TLA** | **I**  **II**  **III**  **IV** | 1.392  1.217  0.782  0.420 | <0.001  <0.001  <0.001  - | **I**  **IIA**  **IIB**  **IIC**  **IIA-C**  **III** | 1.084  2.164  1.837  0.952  1.555  0.297 | <0.001  <0.001  <0.001  <0.001  <0.001  - |

Serum groups used in IgM ELISA test:

I – suspected acute phase of *T. gondii* infection (IgM +; IgG +; low avidity), *n*=48

II – chronic *T. gondii* infection with presence of IgM antibodies (IgM +; IgG +; high avidity), *n*=18

III – chronic *T. gondii* infection with absence of IgM antibodies (IgM –; IgG +; high avidity), *n*=58

IV – control group (IgM –; IgG –), *n*=83
Serum groups used in IgG ELISA test:

I – suspected acute phase of *T. gondii* infection (IgM +; IgG +; low avidity), *n*=64

IIA – chronic *T. gondii* infection (IgM –; IgG >300 IU/ml; high avidity), *n*=32

IIB – chronic *T. gondii* infection (IgM –; IgG 101-300 IU/ml; high avidity), *n*=32

IIC – chronic *T. gondii* infection (IgM –; IgG ≤100 IU/ml; high avidity), *n*=64

IIA-C – chronic *T. gondii* infection (IgM –; IgG +, high avidity), *n*=128

III – control group (IgM –; IgG –), *n*=137
