# Supplementary material for: The first study on the usefulness of recombinant tetravalent chimeric proteins containing fragments of SAG2, GRA1, ROP1 and AMA1 antigens in the detection of specific anti-Toxoplasma gondii antibodies in mouse and human sera
Source: PLoS One. 2019 Jun 6;14(6):e0217866. doi: 10.1371/journal.pone.0217866 (PMC6553757; doi:10.1371/journal.pone.0217866)
Supplement: S3 Table — Serum groups: I–suspected acute phase of T. gondii infection (IgM +; IgG +; low avidity), n = 29 II–chronic T. gondii infection (IgM–; IgG +; high avidity), n = 31 Explanation: AI–avidity index L–low avidity index B–borderline avidity index H–high avidity index For commercial test VIDAS TOXO IgG AVIDITY (bioMérieux, Marcy l'Etoile, France), an AI below 0.2 is low, an AI of 0.2–0.3 is borderline, and an AI over 0.3 is high; for recombinant chimeric proteins and TLA, an AI below 0.3 is low, an AI of 0.3–0.4 is borderline, and an AI over 0.4 is high. (DOCX) [file pone.0217866.s003.docx]

**S3 Table. Comparison of a commercial avidity test and an IgG avidity ELISA tests with recombinant proteins and TLA based on results for sera from patients in the suspected acute (I) or chronic (II) phase of *T. gondii* infection.**

| **ANTIGEN** | **AI: No. (%) of samples** | |
| --- | --- | --- |
|  | **GROUP I**  **(n=29)** | **GROUP II (n=31)** |
| **SAG2-GRA1-ROP1** | L: 26 (89.7) **B: 3 (10.3)**  H: 0 | L: 0 **B: 5 (16.1)**  H: 26 (83.9) |
| **SAG2-GRA1-ROP1-AMA1N** | L: 26 (89.7) **B: 3 (10.3)**  H: 0 | L: 0 **B: 6 (19.4)**  H: 25 (80.6) |
| **AMA1N-SAG2-GRA1-ROP1** | L: 21 (72.4) **B: 8 (27.6)**  H: 0 | L: 0 **B: 2 (6.5)**  H: 29 (93.5) |
| **AMA1C-SAG2-GRA1-ROP1** | L: 17 (58.6) **B: 12 (41.4)**  H: 0 | L: 0 **B: 5 (16.1)**  H: 26 (83.9) |
| **AMA1-SAG2-GRA1-ROP1** | L: 19 (65.5) **B: 10 (34.5)**  H: 0 | L: 0 B: 0  H: 31 (100) |
| **TLA** | L: 20 (69.0) **B: 9 (31.0)**  H: 0 | L: 0 B: 0  H: 31 (100) |

Serum groups:

I – suspected acute phase of *T. gondii* infection (IgM +; IgG +; low avidity), *n*=29

II – chronic *T. gondii* infection (IgM –; IgG +; high avidity), *n*=31

Explanation:

AI – avidity index

L – low avidity index

B – borderline avidity index

H – high avidity index

For commercial test VIDAS TOXO IgG AVIDITY (bioMérieux, Marcy l'Etoile, France), an AI below 0.2 is low, an AI of 0.2-0.3 is borderline, and an AI over 0.3 is high; for recombinant chimeric proteins and TLA, an AI below 0.3 is low, an AI of 0.3-0.4 is borderline, and an AI over 0.4 is high.
