# Supplementary material for: The first study on the usefulness of recombinant tetravalent chimeric proteins containing fragments of SAG2, GRA1, ROP1 and AMA1 antigens in the detection of specific anti-Toxoplasma gondii antibodies in mouse and human sera
Source: PLoS One. 2019 Jun 6;14(6):e0217866. doi: 10.1371/journal.pone.0217866 (PMC6553757; doi:10.1371/journal.pone.0217866)
Supplement: S4 Table — Explanation: IgM: (+)–positive result; (–)–negative result (the result below the cut-off value calculated for the specific antigen preparation)IgG avidity maturation: AI–avidity index; L–low avidity index; B–borderline avidity index; H–high avidity index.* the result includes the repetition resulting from the so-called gray zone IgM, IgG, and IgG avidity maturation on the basis of a ROC analysis. Results marked in bold–incompatible with the results of a commercial test. (DOCX) [file pone.0217866.s004.docx]

**S4 Table. Comparison of IgM, IgG, and IgG avidity maturation of commercial tests results with ELISA’s tests results using recombinant proteins with serum samples obtained from pregnant women suspected of acute phase of *T. gondii* infection taken at various time intervals.**

| **PATIENT** | **NUMBER OF WEEKS** |  | **VIDAS TEST** | **TLA** | **SAG2-GRA1-ROP1** | **SAG2-GRA1-ROP1-AMA1N** | **AMA1N-SAG2-GRA1-ROP1** | **AMA1C-SAG2-GRA1-ROP1** | **AMA1-SAG2-GRA1-ROP** |
| --- | --- | --- | --- | --- | --- | --- | --- | --- | --- |
| **P1** | **0** | IgG  IgM  IgG AI | 127 IU/ml  1.42 (+)  0.117 L | 1.121  1.458 (+)  0.286 L | 1.264  1.064 (+)  0.248 L | 0.588  0.560 (+)  0.267 L | 0.921  1.026 (+)  0.269 L | 1.193  0.730 (+)  0.320 L | 0.896  0.616 (+)  0.226 L |
|  | **7** | IgG  IgM  IgG AI | 130 IU/ml  1.33 (+)  0.114 L | 1.091  1.360 (+)  0.313 L | 1.160  0.885 (+)  0.263 L | 0.659  0.499 (+)  0.291 L | 0.831  0.957 (+)  0.281 L | 0.898  0.877 (+)  0.338 L | 0.691  0.740 (+)  0.254 L |
|  | **23** | IgG  IgM  IgG AI | 107 IU/ml  0.94 (+)  0.154 L | 0.863  1.070 (+)  0.333 L | 0.825  0.918 (+)  0.271 L | 0.417  0.580 (+)  **0.320 B** | 0.533  0.868 (+)  0.305 L | 0.561  0.853 (+)  **0.366 B** | 0.646  0.793 (+)  0.336 L |
| **P2** | **0** | IgG  IgM  IgG AI | 278 IU/ml  1.46 (+)  0.160 L | 1.225  1.117 (+)  0.245 L | 2.004  **0.446 (-)***  0.216 L | 0.772  0.354 (+)  0.234 L | 1.505  **0.460 (-)***  0.252 L | 1.576  0.457 (+)  0.299 L | 1.714  0.515 (+)  0.255 L |
|  | **6** | IgG  IgM  IgG AI | 179 IU/ml  1.40 (+)  0.145 L | 1.134  1.138 (+)  0.271 L | 1.553  0.532 (+)  0.237 L | 0.573  0.334 (+)  0.270 L | 1.081  0.529 (+)  0.255 L | 1.033  **0.376 (-)***  0.298 L | 1.289  0.547 (+)  0.288 L |
| **P3** | **0** | IgG  IgM  IgG AI | 201 IU/ml  1.23 (+)  0.140 L | 1.224  1.207 (+)  0.291 L | 1.836  0.544 (+)  0.262 L | 1.144  **0.303 (-)***  0.204 L | 1.331  0.502 (+)  0.285 L | 1.437  0.526 (+)  0.290 L | 1.331  0.418 (+)  0.292 L |
|  | **5** | IgG  IgM  IgG AI | 180 IU/ml  1.31 (+)  0.148 L | 1.399  1.280 (+)  **0.382 B** | 1.846  0.519 (+)  **0.316 B** | 1.177  **0.254 (-)***  0.250 L | 1.415  0.527 (+)  0.340 L | 1.407  **0.404 (-)***  0.316 L | 1.440  0.369 (+)  0.316 L |
|  | **10** | IgG  IgM  IgG AI | 259 IU/ml  1.34 (+)  0.195 L | 1.424  1.156 (+)  **0.390 B** | 1.693  0.467 (+)  **0.318 B** | 1.163  0.346 (+)  0.254 L | 1.412  **0.453 (-)***  **0.364 B** | 1.508  0.486 (+)  **0.358 B** | 1.459  0.506 (+)  0.330 L |
|  | **15** | IgG  IgM  IgG AI | 238 IU/ml  1.31 (+)  0.175 L | 1.603  1.301 (+)  **0.397 B** | 1.981  0.467 (+)  **0.380 H** | 1.370  0.326 (+)  **0.342 B** | 1.576  0.526 (+)  **0.375 B** | 1.644  0.555 (+)  **0.380 B** | 1.699  0.520 (+)  0.358 L |
| **P4** | **0** | IgG  IgM  IgG AI | 91 IU/ml  2.70 (+)  0.139 L | 0.829  1.688 (+)  0.257 L | 1.169  1.686 (+)  0.240 L | 0.743  1.424 (+)  0.218 L | 0.942  1.676 (+)  0.273 L | 0.915  1.496 (+)  0.243 L | 0.977  1.646 (+)  0.259 L |
|  | **11** | IgG  IgM  IgG AI | 63 IU/ml  2.80 (+)  0.159 L | 0.913  1.526 (+)  0.268 L | 1.160  1.449 (+)  0.298 L | 0.633  1.206 (+)  0.232 L | 0.840  1.379 (+)  0.283 L | 0.989  1.255 (+)  0.271 L | 0.857  1.431 (+)  0.285 L |
|  | **15** | IgG  IgM  IgG AI | 90 IU/ml  2.69 (+)  0.150 L | 1.041  1.475 (+)  0.269 L | 1.212  1.062 (+)  0.289 L | 0.744  1.115 (+)  0.248 L | 0.871  1.257 (+)  **0.370 B** | 1.082  1.363 (+)  0.330 L | 0.928  1.310 (+)  0.303 L |

Explanation:

- IgM: (+) – positive result; (–) – negative result (the result below the cut-off value calculated for the specific antigen preparation)
- IgG avidity maturation: AI – avidity index; L – low avidity index; B – borderline avidity index; H – high avidity index.
- * the result includes the repetition resulting from the so-called gray zone

IgM, IgG, and IgG avidity maturation on the basis of a ROC analysis.

Results marked in bold – incompatible with the results of a commercial test.
